# Loss of the Essential Autophagy Regulators FIP200 or Atg5 Leads to Distinct Effects on Focal Adhesion Composition and Organization

**DOI:** 10.3389/fcell.2020.00733

**Published:** 2020-08-04

**Authors:** Emelia A. Assar, David A. Tumbarello

**Affiliations:** Biological Sciences, University of Southampton, Southampton, United Kingdom

**Keywords:** focal adhesion kinase, Src, cell adhesion, actin cytoskeleton, autophagy

## Abstract

Autophagy is an essential catabolic intracellular pathway that maintains homeostasis by degrading long-lived proteins, damaged organelles, and provides an energy source during nutrient starvation. It is now understood that autophagy has discrete functions as a selective lysosomal degradation pathway targeting large cytosolic structural and signaling complexes to influence cell motility and adhesion. We provide evidence suggesting the primary autophagy regulators Atg5 and FIP200 both play a role in cell motility and extracellular matrix adhesion. However, their loss of function has a differential impact on focal adhesion composition and organization, as well as signaling in response to fibronectin induced cell spreading. This differential impact on focal adhesions is illustrated by smaller focal adhesion complexes and a decrease in FAK, paxillin, and vinculin expression associated with FIP200 loss of function. In contrast, Atg5 loss of function results in production of large and stable focal adhesions, characterized by their retention of phosphorylated FAK and Src, which correlates with increased vinculin and FAK protein expression. Importantly, autophagy is upregulated during processes associated with focal adhesion reorganization and their exhibits colocalization of autophagosomes with focal adhesion cargo. Interestingly, FIP200 localizes to vinculin-rich focal adhesions and its loss negatively regulates FAK phosphorylation. These data collectively suggest FIP200 and Atg5 may have both autophagy-dependent and -independent functions that provide distinct mechanisms and impacts on focal adhesion dynamics associated with cell motility.

## Introduction

The autophagy pathway is essential to maintain cellular homeostasis by facilitating the removal of aggregated or long-lived proteins, damaged organelles, and invading pathogens, as well as acting as an energy source during nutrient deprivation. However, it is now understood that autophagy can selectively target discrete protein complexes to regulate cell function (Gao et al., 2010; Sandilands et al., 2012a). Proteins such as the non-receptor tyrosine kinase Src, Ret tyrosine kinase, the Wnt signaling regulator Disheveled, and the focal adhesion protein paxillin have all been identified to be directly targeted by autophagosomes in order to facilitate their turnover in the lysosome (Gao et al., 2010; Sandilands et al., 2012a, b; Sharifi et al., 2016). Importantly, this is likely context dependent, triggered by acute and specific stimuli that require engagement with and activation of primary autophagy regulators in order to trigger formation of an autophagosome around each specified cargo.

Cell motility requires the coordinated reorganization of focal adhesion (FA) complexes and the actin cytoskeleton to facilitate movement along the extracellular matrix (ECM). Focal adhesions are comprised of both structural components that link the integrin receptor mediated ECM attachment points to the actin cytoskeleton, providing biomechanical force and tensile strength, as well as signaling proteins that trigger a range of responses that ensure dynamic regulation of the actin cytoskeleton and focal adhesions during cell motility (Kuo, 2013). It has been suggested that autophagy directly regulates focal adhesion dynamics by facilitating the targeted degradation of key signaling and adaptor proteins (Sandilands et al., 2012a; Kenific et al., 2016a; Sharifi et al., 2016). More generally, this selective form of autophagy is mediated by the ubiquitylation of discrete cargo that subsequently recruits autophagy receptors, such as NBR1, optineurin, NDP52, tax1bp1, or p62, which contain conserved ubiquitin binding domains that recognize distinct ubiquitin species and specified LC3-interacting regions (LIRs) that interact with autophagosome membrane (Pankiv et al., 2007; Kirkin et al., 2009; Von Muhlinen et al., 2010; Wild et al., 2011; Tumbarello et al., 2015). This results in the encapsulation of cargo within an autophagosome, followed by their transport to the lysosome for degradation. It is thought that this selective process for the targeted turnover of focal adhesion components helps facilitate coordinated cell movement (Kenific et al., 2016b; Sharifi et al., 2017).

One of the major regulators of autophagy is Atg5, which when in complex with Atg12 is essential for the lipidation of Atg8 family members, such as LC3, and their insertion into the growing autophagosome (Hanada et al., 2007). When cells are deficient of Atg5, autophagosomes are unable to form, despite the elongation of an isolation membrane (Kishi-Itakura et al., 2014). Interestingly, genetic disruption of autophagy, by specifically targeting Atg5 or Atg7, results in a modulation of focal adhesion organization impacting on ECM-induced cell spreading (Kawano et al., 2017). It has been suggested that autophagosomes target directly to focal adhesions and are more closely associated with focal adhesion disassembly (Kenific et al., 2016a). This is consistent with mechanisms of podosome disassembly, which results from the autophagy-dependent degradation of Kindlin3 to stimulate inactivation of integrin receptors (Zhang et al., 2020). In addition, it is thought that focal adhesion disassembly is primarily mediated by the autophagy receptor NBR1, which is targeted to focal adhesions to provide the selectivity toward discrete focal adhesion cargo. It has additionally been suggested that the E3 ubiquitin ligase c-cbl, which contains an LIR motif, can selectively target specific FA components, such as active Src for degradation via autophagy to regulate cancer cell viability (Sandilands et al., 2012a). Furthermore, the Ret tyrosine kinase is also sequestered away from focal adhesions and degraded by autophagy in a Src-dependent manner (Sandilands et al., 2012b). Interestingly, these mechanisms of Src regulation are functioning when the activity of focal adhesion kinase (FAK) is low or absent (Sandilands et al., 2012a).

FAK-family Interacting Protein of 200 kDa (FIP200) was initially identified to directly interact with the FA components FAK and the related member Pyk2 to regulate their activity, subsequently influencing cell growth, proliferation, and motility (Ueda et al., 2000; Abbi et al., 2002). However, FIP200 was later identified as an interacting partner of the serine/threonine kinase ULK1, acting as a primary regulator of autophagosome formation as part of the ULK initiation complex (Hara et al., 2008). The ULK1 complex is comprised of ULK1, FIP200, Atg13, and Atg101, which acts to facilitate phagophore formation through activation of the Beclin1-VPS34 initiation complex, which, following the generation of PI3P, triggers the recruitment of key autophagy regulators involved in autophagosome membrane formation and expansion (Zachari and Ganley, 2017). Although *in vivo* studies have illustrated that FIP200 performs both autophagy-dependent and -independent functions (Chen et al., 2016), little is currently known about the mechanisms that drive this selectivity and whether some of these functions overlap to regulate these disparate cellular processes.

Therefore, we aimed to understand whether targeting distinct autophagy regulators would differentially impact on cell motility and focal adhesion organization due to their divergent autophagy-independent roles (Galluzzi and Green, 2019). We set out to perform this analysis using CRISPR-Cas9 generated knockouts in a breast epithelial cell line, which allowed us to carefully dissect impacts on focal adhesion composition and organization in the context of cell motility. Our results indicate that loss of Atg5 and FIP200 both negatively impact on cell motility and enhance fibronectin-induced adhesion, but have differential impacts on focal adhesion composition, organization, and dynamics. Thus, our data suggests both autophagy-dependent and -independent functions of key autophagy initiators exists to regulate focal adhesion dynamics during cell motility.

## Results

### Depletion of FIP200 or Atg5 From Breast Epithelial Cells Reduces Directional Cell Motility

**FIGURE 1:**
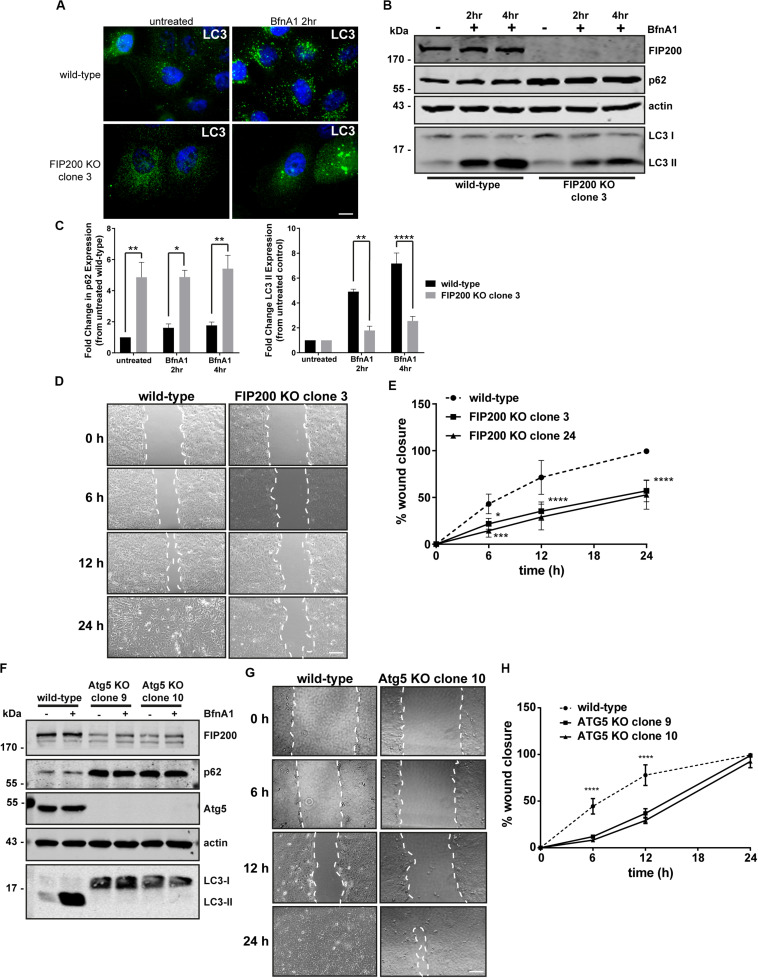
Loss of the autophagy regulators FIP200 or Atg5 results in inhibition of directional cell motility. **(A)** Wild-type and FIP200 knockout (KO) MCF10A cells processed for immunofluorescence microscopy and immunostained for LC3 (green). Nuclei are labeled with Hoechst (blue). Scale bar = 10 μm. **(B)** Western blot analysis on lysates harvested from wild-type and FIP200 KO MCF10A cells either left untreated or treated with Bafilomycin A1 (BfnA1) for 2 and 4 hours. Immunoblotting was performed against the indicated proteins. **(C)** Quantitation of western blot data illustrating the fold change in p62 and LC3-II levels in untreated and Bafilomycin treated cells. Levels of p62 and LC3-II were normalized to actin and represented as fold change from untreated wild-type control cells. Results represent at least 3 independent experiments and error bars are SEM. **(D)** MCF10A wild-type and FIP200 KO cells were subjected to a scratch wound assay and brightfield images were taken at initial wounding (0 h) and at 6, 12, and 24 hours post-wounding. Dotted white lines highlight wound edge. Scale bar = 200 μm. **(E)** Quantitation of scratch wound assay was performed and represented as % of wound closure, calculated as a percentage of total area of initial wound at 0 h. Results represent at least 3 independent experiments and errors bars illustrate SEM. **(F)** Western blot analysis on lysates harvested from wild-type and two clones of Atg5 KO MCF10A cells either left untreated or treated with BfnA1 for 2 and 4 hours. Immunoblotting was performed against the indicated proteins. **(G)** MCF10A wild-type and Atg5 KO cells were subjected to a scratch wound assay and brightfield images were taken at initial wounding (0 h) and at 6, 12, and 24 hours post-wounding. Dotted white lines highlight wound edge. Scale bar = 200 μm. **(H)** Quantitation of scratch wound assay was performed and represented as % of wound closure, calculated as a percentage of total area of initial wound at 0 h. Results represent at least 3 independent experiments and errors bars illustrate SEM. *p < 0.05, **p < 0.01, ***p < 0.001, ****p < 0.0001.

FIP200 has been described as a direct regulator of both focal adhesion signaling (Ueda et al., 2000; Abbi et al., 2002) and autophagosome formation (Hara et al., 2008). It is also now widely understood that autophagy plays a direct role in modulating focal adhesion composition and signaling (Sandilands et al., 2012a; Kenific et al., 2016a; Sharifi et al., 2016). Therefore, we initially aimed to develop a FIP200 knockout (KO) model in a breast epithelial cell line to investigate whether its loss of function modulates cell motility, as a result of its association with focal adhesion regulation. We developed two independent CRISPR-Cas9 clonal lines from MCF10A cells that were successfully depleted of FIP200 protein expression and exhibited a defect in autophagy function (Figures 1A–C and Supplementary Figure S1). The autophagy deficiency in FIP200 KO cells was characterized by elevated p62 expression and a reduction in LC3-II expression following Bafilomycin A1 (BfnA1) treatment (Figures 1B,C and Supplementary Figure S1), suggesting at least a partial defect in autophagosome formation. However, it should be noted that although FIP200 KO cells did retain a partial level of LC3 lipidation, illustrated by an accumulation of LC3-II expression in response to BfnA1 by western blot (Figure 1B and Supplementary Figure S1), this was characterized by an atypical LC3 localization shown by microscopy (Figure 1A), which appears as large, swollen aggregates. These data may suggest either mis-targeting of LC3 to single membrane structures or the presence of FIP200-independent mechanisms of autophagy at steady state (Cheong et al., 2011; Florey et al., 2011). We next evaluated the directional motility of two separate FIP200 KO clones using a scratch wound assay. Our results indicate that both FIP200 KO clonal lines exhibit reduced scratch wound closure, indicating a defect in directional cell motility (Figures 1D,E).

Due to residual LC3 lipidation in the FIP200 KO cell lines and the alternative function of FIP200 to regulate focal adhesion signaling via direct interactions with FAK and Pyk2 (Ueda et al., 2000; Abbi et al., 2002), we took the approach to deplete cells of Atg5, a primary autophagy regulator, in order to understand the potential contribution of bulk autophagy to our initial findings. We therefore created two distinct Atg5 KO clonal lines, using CRISPR-Cas9, which exhibited elevated p62 expression and a complete loss of LC3-II expression by western blot (Figure 1F), indicating a block in autophagosome formation. Interestingly, both Atg5 KO clones exhibited a significant reduction in FIP200 expression, while in contrast FIP200 KO clones exhibited a marginal increase in Atg5 expression (Supplementary Figure S2). Although the Atg5 KO cell lines demonstrated a defect in directional motility indicated by a reduction in wound closure at 6 and 12 hours post-wounding, these cells were able to recover to close the wounds at 24 hours, in contrast to FIP200 KO cells (Figures 1G,H). Therefore, although loss of autophagy by targeting two independent gene targets reduced directional cell motility in MCF10A cells, depletion of FIP200 had a more pronounced effect on this process, suggesting potential non-redundant functions of these two proteins.

### Autophagy Is Induced During Cell Spreading and Its Perturbation Increases the Extent of Cell Spread

**FIGURE 2:**
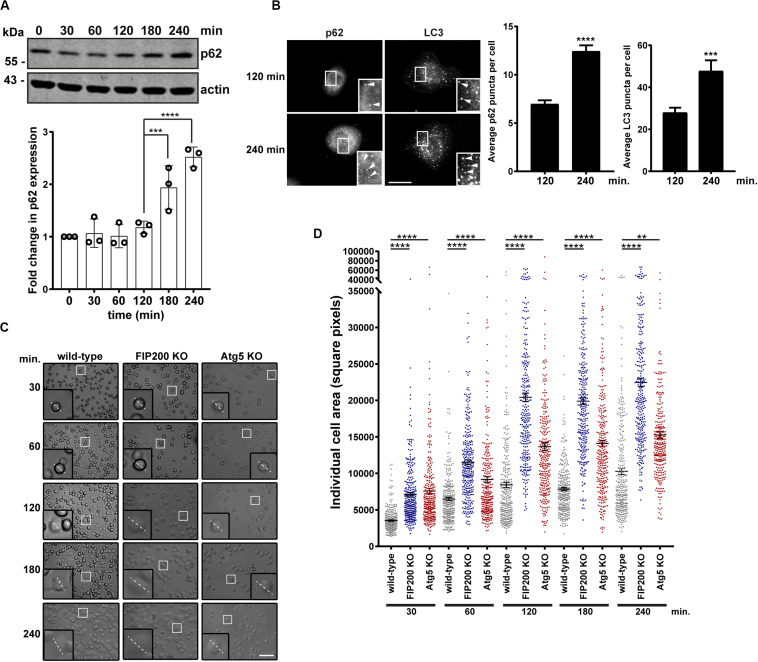
FIP200 or Atg5 depletion increases fibronectin-induced cell adhesion and spreading. **(A)** Western blot analysis of cell lysates harvested along the time course of fibronectin-induced MCF10A wild-type cell spreading. Immunoblotting was performed against the indicated proteins. Quantitation of p62 expression was normalized to actin and represented as the fold change from the zero time point (cells in suspension). Results represent at least 3 independent experiments. **(B)** Immunofluorescence microscopy of MCF10A cells following attachment and spreading on fibronectin for indicated time points. Cells were immunostained for p62 and LC3. Scale bar = 10 μm. Quantitation was performed on multiple images from random fields of view. The number of p62 or LC3 puncta (indicated with arrowheads) were quantified from 100 cells for p62 and 13–15 cells for LC3 across 3 independent experiments. Results represent the average number of p62 or LC3 puncta per cell. Error bars represent SEM. **(C)** Brightfield images across the time course of adhesion to fibronectin of MCF10A wild-type, FIP200 KO, and Atg5 KO cells. Higher magnification regions illustrate phenotypic differences and dotted line represents representative cell diameter. Scale bar = 100 μm. **(D)** Quantitation of individual cell area represented as square pixels were plotted across the time course of fibronectin adhesion. Results represent 50–100 cells per experiment, repeated at least 3 independent times. Black bar represents the mean and error bars are the SEM. **p < 0.01, ***p < 0.001, ****p < 0.0001.

Directional cell motility requires polarized membrane protrusion, which is accompanied by focal adhesion formation to stabilize cell-ECM contact at the leading edge, while random migration lacks polarity and therefore membrane protrusions extend in all directions (Ridley et al., 2003; Petrie et al., 2009). Following our analysis of directional cell motility, where there were defects in both FIP200 and Atg5 KO cells, we next evaluated random membrane protrusion using a fibronectin-induced cell spreading assay. We first set out to determine whether autophagy is active during fibronectin-induced cell spreading. Following MCF10A cell attachment and spreading on fibronectin, autophagy is induced as illustrated by an accumulation of the autophagy receptor p62 by western blot (Figure 2A). We next evaluated, by immunofluorescence microscopy, both p62 and LC3 puncta over the time course of the spreading process to confirm whether autophagy is induced. These data indicate a clear increase of both p62 and LC3 spots, indicating autophagosomes, within the cytosol of cells at 240 min post-fibronectin induced spreading (Figure 2B). We next aimed to determine whether loss of FIP200 and Atg5 expression, which resulted in decreased directional cell motility, was due to a decrease in membrane protrusion. We therefore performed fibronectin-induced spreading assays with FIP200 and Atg5 KO cells. Indeed, loss of both FIP200 and Atg5 similarly resulted in increased cell spreading at each of the time points following fibronectin attachment, which is illustrated by brightfield imaging and following quantitation of cell area (Figures 2C,D). Therefore, these data indicate that cells lacking FIP200 or Atg5 expression, while showing a decrease in directional motility, are capable of forming membrane protrusions and spread on fibronectin at a faster rate compared to wild-type cells.

**FIGURE 3:**
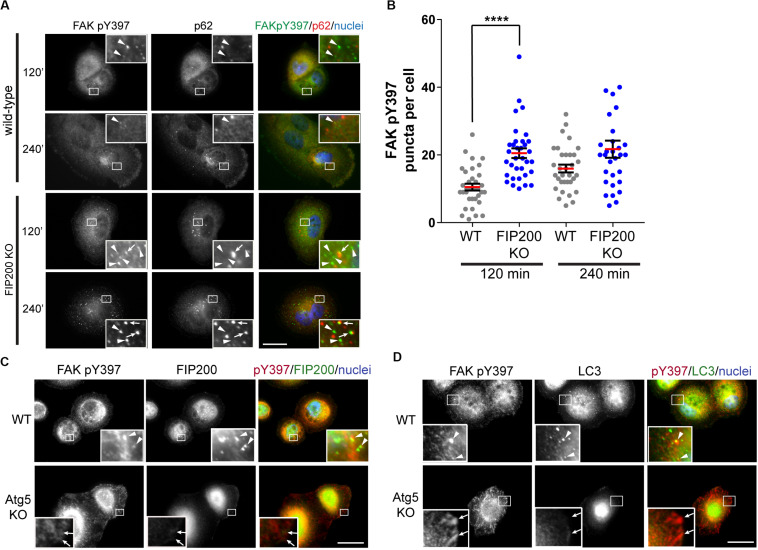
Differential dysregulation of FAK pY397 trafficking in FIP200 and Atg5 KO cells. **(A)** MCF10A wild-type and FIP200 KO cells were subjected to fibronectin induced cell adhesion and fixed at indicated time points of 120 and 240 minutes post-plating. Immunostaining was performed against FAK pY397 (green) and p62 (red). Nuclei were labeled with Hoechst (blue). Arrowheads indicate FAK pY397 spots and arrows indicate FAK pY397 and p62 colocalization. **(B)** Quantitation of immunofluorescence images taken from cells subjected to fibronectin induced adhesion. The number of FAK pY397 puncta per cell were quantified from between 30 and 34 cells per group across random fields of view from 2 independent experiments. Each data point indicates a cell, the red bar indicates mean value and error bars represent SEM. ****p < 0.0001. Immunofluorescence microscopy performed on wild-type (WT) and Atg5 KO MCF10A cells following attachment to fibronectin. Cells were fixed at 120 min post-plating and immunostained in **(C)** for FAK pY397 (red) and FIP200 (green), and in **(D)** for FAK pY397 (red) and LC3 (green). Nuclei are labeled with Hoechst (blue). Arrowheads indicate areas of colocalization and arrows indicate FA-like immunostaining. Scale bars = 10 μm.

As a result of previous reports indicating FIP200 directly modulates the activation state of FAK (Abbi et al., 2002), we next evaluated the subcellular localization of the tyrosine phosphorylated form of FAK (FAK pY397) during fibronectin induced adhesion and spreading. During MCF10A cell spreading on fibronectin, an increase in FAK pY397 puncta was exhibited, which was potentiated following loss of FIP200 expression (Figures 3A,B). Interestingly, a subset of these FAK pY397 spots colocalized with the autophagy receptor p62 (Figure 3A), which recognizes ubiquitylated cargo and is itself degraded by autophagy (Bjorkoy et al., 2005). In addition, a small proportion of these FAK pY397 spots colocalized with FIP200 and LC3 in wild-type cells (Figures 3C,D), while FAK pY397 colocalization with LC3-positive autophagosomes are clearly absent in Atg5 KO cells. In contrast to FIP200 depletion, loss of Atg5 resulted in a retention of FAK pY397 at focal adhesions (Figures 3C,D) suggesting a defect in focal adhesion dynamics during cell spreading on fibronectin following bulk autophagy inhibition.

### Loss of FIP200 Results in Increased ERK1 and ERK2 Phosphorylation in Response to Adhesion and Spreading on Fibronectin

**FIGURE 4:**
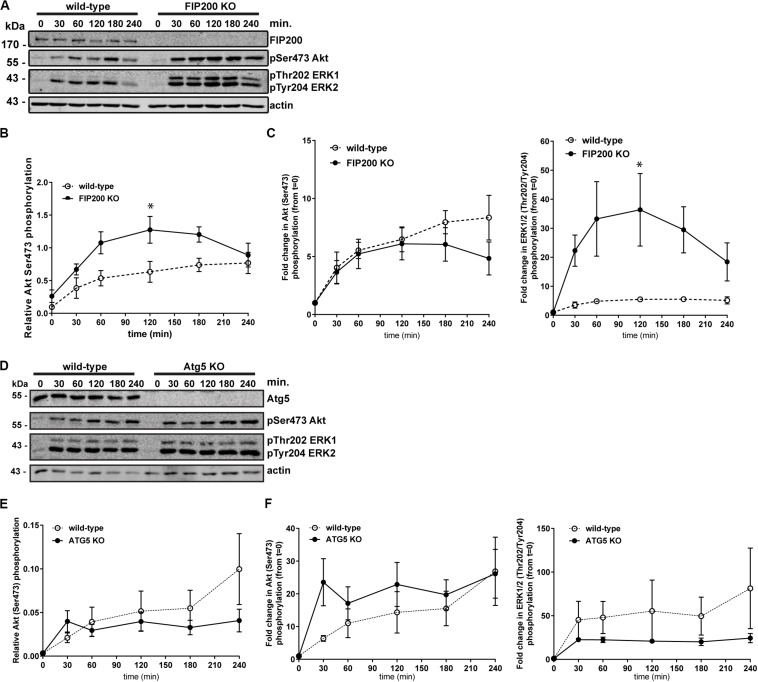
Specific loss of FIP200 results in elevated phospo-ERK1/2 across the time course of fibronectin-induced cell spreading. **(A)** MCF10A wild-type and FIP200 KO cells were kept in suspension in EGF depleted growth media for 1 hour prior to plating on 10 μg/ml fibronectin for the indicated times. Time zero indicates cells in suspension. Western blot analysis was performed on lysates harvested at the indicated times. Immunoblotting was performed against the indicated proteins. **(B)** Quantitation of Ser473 Akt phosphorylation western blot integrated density values normalized to actin from wild-type and FIP200 KO cells. **(C)** Fold change in Akt Ser473 or ERK1 Thr202 and ERK2 Tyr204 phosphorylation from zero time point following normalization to actin from wild-type and FIP200 KO cells. **(D)** MCF10A wild-type and Atg5 KO cells were kept in suspension in EGF depleted growth media for 1 hour prior to plating on 10 μg/ml fibronectin for the indicated times. Time zero indicates cells in suspension. Western blot analysis was performed on lysates harvested at the indicated times. Immunoblotting was performed against the indicated proteins. **(E)** Quantitation of Ser473 Akt phosphorylation western blot integrated density values normalized to actin from wild-type and Atg5 KO cells. **(F)** Fold change in Akt Ser473 or ERK1 Thr202 and ERK2 Tyr204 phosphorylation from zero time point following normalization to actin from wild-type and Atg5 KO cells. Results represent at least 3 independent experiments and error bars are the SEM. *p < 0.05.

We next aimed to further investigate the potential mechanisms of Atg5 and FIP200 function during cell spreading. We therefore evaluated downstream signaling events, specifically measuring Akt and ERK1/2 phosphorylation along the time course of fibronectin induced spreading in FIP200 and Atg5 KO cells. Interestingly, loss of FIP200 resulted in a substantial increase in phosphorylation of ERK1 and ERK2 in response to fibronectin-induced cell spreading (Figures 4A–C). While FIP200 KO cells have elevated phosho-Ser473 Akt levels, there is no significant difference from wild-type in their response to fibronectin (Figures 4A–C). In contrast, Atg5 KO cells do not exhibit elevated ERK phosphorylation and instead trend lower than wild-type in response to fibronectin. In addition, no significant differences in Akt phosphorylation from wild-type are observed (Figures 4D–F). These data suggest loss of FIP200 expression has distinct impacts on signaling induced by adhesion to fibronectin.

### Loss of FIP200 or Atg5 Have Contrasting Effects on Focal Adhesion Composition

**FIGURE 5:**
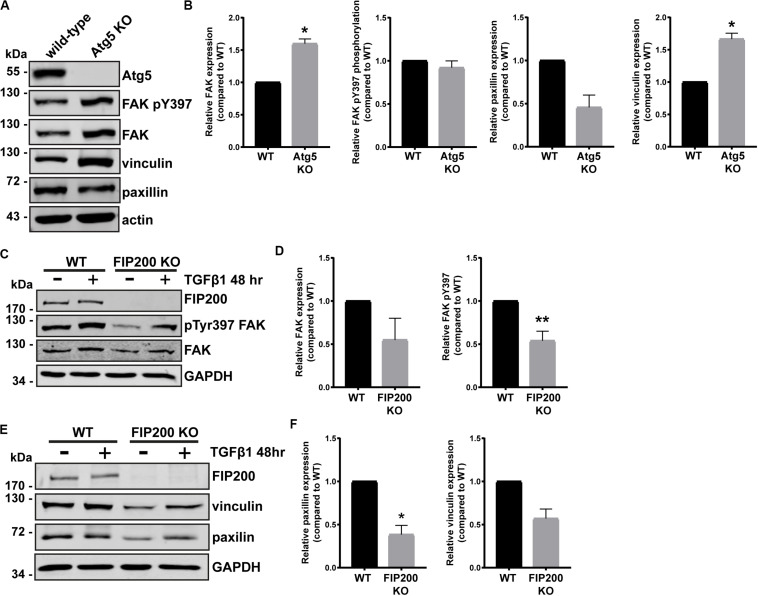
FIP200 or Atg5 depletion has a differential impact on focal adhesion composition. **(A)** Western blot analysis of lysates harvested from MCF10A wild-type and Atg5 KO cells cultured in normal growth media. Immunoblotting performed on indicated proteins. **(B)** Quantitation of relative FAK, FAK pY397, paxillin, and vinculin levels. Graphs represent levels compared to wild-type and are comprised of at least 3 independent experiments. **(C)** Western blot analysis of lysates harvested from MCF10A wild-type and FIP200 KO cells either untreated or treated with 5 ng/ml TGFβ1 in normal growth media for 48 hours. Immunoblotting was performed against the indicated proteins. **(D)** Quantitation of relative FAK and FAK pY397 levels. Graphs represent levels compared to wild-type and are comprised of at least 3 independent experiments. **(E)** Western blot analysis of lysates harvested from MCF10A wild-type and FIP200 KO cells either untreated or treated with 5 ng/ml TGFβ1 in normal growth media for 48 hours. Immunoblotting was performed against the indicated proteins. **(F)** Quantitation of relative paxillin and vinculin expression was performed. Graphs represent levels compared to wild-type and are comprised of at least 3 independent experiments. *p < 0.05, **p < 0.01.

Although FIP200 and Atg5 loss of function similarly result in decreased directional cell motility and increased cell spreading, there were distinct effects on intracellular signaling events. We therefore wanted to determine whether loss of either impacted on focal adhesion composition, by probing for key regulators of cell-ECM attachment and focal adhesion signaling. Loss of Atg5, which specifically disrupts the autophagy pathway resulted in a significant accumulation of vinculin and FAK protein expression, while paxillin expression was reduced compared to wild-type (Figures 5A,B). However, there was no effect on FAK pY397 levels (Figures 5A,B). We next evaluated the composition of focal adhesions in FIP200 KO MCF10A cells both in the epithelial state and following TGFβ1 induced epithelial-mesenchymal transition (EMT). It is well-established that the process of EMT promotes the reorganization of the actin cytoskeleton, leading to cell-ECM adhesion and focal adhesion assembly (Yilmaz and Christofori, 2009). Interestingly, in epithelial MCF10A cells, loss of FIP200 resulted in decreased FAK, paxillin, and vinculin expression (Figures 5C–F), while also exhibiting a significant suppression in FAK pY397 levels (Figures 5C,D). In the absence of FIP200, MCF10A cells still retain a response to TGFβ1 induced EMT exhibited by increased FAK pY397, although this is suppressed compared to wild-type (Figure 5C). These results indicate that loss of FIP200 and Atg5 expression have significant, but contrasting, effects on focal adhesion composition.

### FIP200 KO Cells Can Assemble Vinculin-Positive Focal Adhesions, While Exhibiting an Impairment in Focal Adhesion Disassembly

**FIGURE 6:**
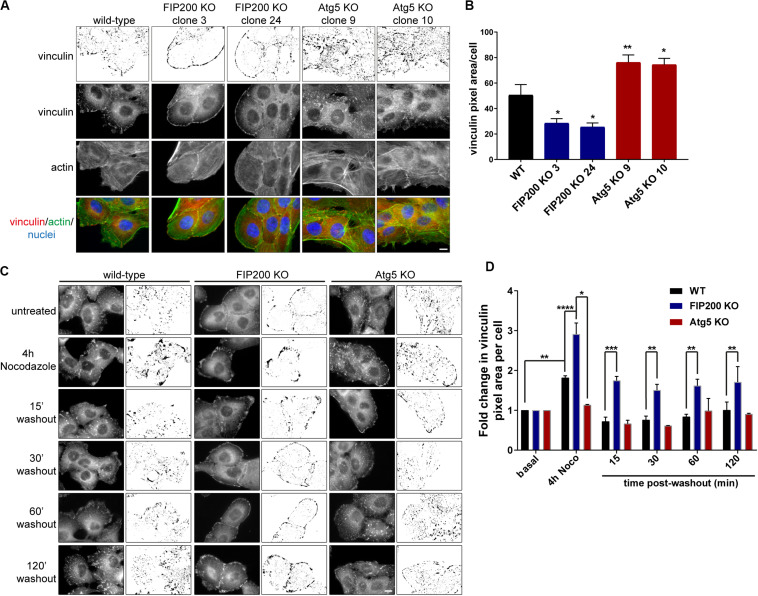
Loss of FIP200 or Atg5 has a differential effect on focal adhesion organization and dynamics. **(A)** Immunofluorescence microscopy of MCF10A wild-type, two FIP200 KO clones, and two Atg5 KO clones from asynchronous cells cultured in normal growth media was performed against vinculin (green) and actin (red), visualized with phalloidin. Nuclei are labeled with Hoechst (blue). Top panel shows inverted vinculin immunostaining following background subtraction, in order to highlight variation in focal adhesion organization. **(B)** Quantitation of vinculin immunostaining was performed. Graph represents average vinculin pixel area per cell. Data is comprised of at least 5–10 cells/experiment from random fields of view across 2 independent experiments. **(C)** Nocodazole assay of MCF10A wild-type, FIP200 KO, and Atg5 KO cells. Cells were either left untreated or treated with Nocodazole for 4 hours in serum-free media prior to washout of Nocodazole and recovery in growth media for indicated time points. Cells were fixed and immunostained for vinculin at each time point. Images are shown as raw monochrome images and inverted images following background subtraction to highlight vinculin focal adhesion staining. **(D)** Quantitation was performed on vinculin images following background subtraction and represented as the fold change in vinculin pixel area per cell from untreated control. Results represent 3 independent experiments for wild-type and FIP200 KO cells and 2 independent experiments for Atg5 KO cells. *p < 0.05, **p < 0.01, ***p < 0.001, ****p < 0.0001. Scale bars = 10 μm.

In order to understand the differences in focal adhesion composition between FIP200 and Atg5 KO cells, we first employed immunofluorescence microscopy to observe their effects on focal adhesion organization. These data illustrate that compared to wild-type MCF10A cells, FIP200 KO cells have much smaller focal contacts identified by vinculin that are arranged along the cell periphery, which correlates with a cortical actin distribution. Further analysis of FIP200 KO vinculin-positive focal adhesions reveals a reduced pixel area per cell (Figures 6A,B). In contrast, Atg5 KO MCF10A cells have much larger vinculin-positive focal adhesions scattered throughout the cell that cover a greater cellular area than wild-type, which correlates with more robust actin stress fibers traversing the cell (Figures 6A,B).

We next aimed to understand the impact of Atg5 or FIP200 loss of function on focal adhesion dynamics. To do these analyses, we employed a Nocodazole washout assay (Ezratty et al., 2005), which allowed us to evaluate both focal adhesion assembly and disassembly, as indicated by vinculin staining, in a synchronized manner. MCF10A cells were serum-starved prior to treatment with 10 μM Nocodazole for 4 hours, which destabilizes the microtubules leading to robust focal adhesion assembly (Figures 6C,D). Following this, Nocodazole was washed away from cells and replenished with fresh growth media to allow for microtubule regrowth and stimulation of focal adhesion disassembly (Figures 6C,D). However, in the absence of Atg5, microtubule destabilization does not lead to an increase in vinculin area, nor do we see any noticeable disassembly upon nocodazole washout. These data illustrate that Atg5 KO cells exhibit very stable, mature focal adhesions, similar to previous published reports (Kawano et al., 2017). In contrast, loss of FIP200 leads to significant focal adhesion assembly following microtubule destabilization, but these vinculin-positive adhesions are unable to disassemble back to steady state levels following microtubule regrowth (Figures 6C,D). Collectively, these data suggest that Atg5 and FIP200 have distinct mechanisms to regulate focal adhesion organization and dynamics, suggesting a potential contribution from autophagy-independent functions to regulate these processes.

### FIP200 Localizes to Vinculin-Positive Focal Adhesions, With Limited Localization to LC3 Positive Autophagosomes

**FIGURE 7:**
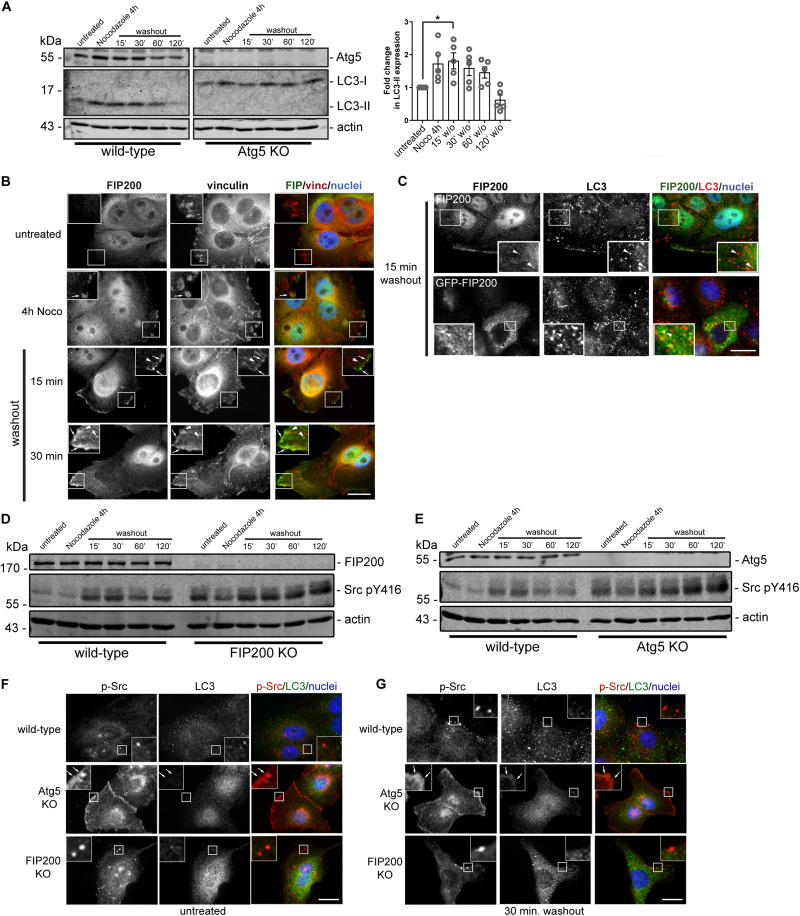
Autophagy is activated during focal adhesion reorganization, which correlates with FIP200 localization to focal adhesions and autophagosomes. **(A)** Western blot analysis was performed on lysates harvested from MCF10A wild-type and Atg5 KO cells along the time course of the Nocodazole assay. Cells were either left untreated or treated with Nocodazole for 4 hours in serum-free media prior to washout of Nocodazole and recovery in growth media for indicated time points. Immunoblotting was performed against the indicated proteins and fold change in LC3-II levels were quantitated. Graph represents the fold change in LC3-II from untreated control following normalization to actin. One way ANOVA followed by Dunnett’s multiple comparison test was performed, *p < 0.05. Error bars represent the SEM. **(B)** Immunofluorescence microscopy of FIP200 (green) and vinculin (red) colocalization along selected time points of the Nocodazole assay. Nuclei are labeled with Hoechst (blue). Arrows indicate FIP200 FA-like immunostaining and arrowheads indicate FIP200 vesicular localization. **(C)** Immunofluorescence microscopy of either endogenous FIP200 or GFP-FIP200 (green) and LC3 (red) in MCF10A cells at 15 min post-Nocodazole washout. Nuclei are labeled with Hoechst (blue). Arrowheads indicate areas of colocalization. **(D)** Western blot analysis on cell lysates harvested from MCF10A wild-type and FIP200 KO cells treated as indicated across the Nocodazole washout assay. Cells were either left untreated or treated with Nocodazole for 4 hours in serum-free media prior to washout of Nocodazole and recovery in growth media for indicated time points. Antibodies against the specified proteins are indicated. **(E)** Western blot analysis on cell lysates harvested from MCF10A wild-type and Atg5 KO cells treated as indicated across the Nocodazole washout assay. Cells were either left untreated or treated with Nocodazole for 4 hours in serum-free media prior to washout of Nocodazole and recovery in growth media for indicated time points. Antibodies against the specified proteins are indicated. Immunofluorescence microscopy was performed on MCF10A wild-type, FIP200 KO, and Atg5 KO untreated cells **(F)** or after 30 min washout following Nocodazole treatment **(G)**. Cells were immunostained for pY416 Src (p-Src) (red) and LC3 (green). Nuclei were labeled with Hoechst (blue). Arrows indicate FA-like immunostaining. Scale bars = 10 μm.

We next aimed to determine whether, during the time course of focal adhesion assembly and disassembly, autophagy is active. We therefore performed western blot analysis on lysates harvested from MCF10A cells during the time course of the Nocodazole washout assay. Interestingly, LC3-II accumulates during focal adhesion assembly associated with microtubule destabilization and stays elevated following Nocodazole washout at 15 minutes, before decreasing at later time points during focal adhesion disassembly (Figure 7A). To confirm the specificity, we used Atg5 KO cells to illustrate the complete loss of LC3-II expression over the course of the Nocodazole washout assay (Figure 7A). We next evaluated FIP200 localization during focal adhesion assembly and disassembly to understand its spatial organization during these processes. Interestingly, FIP200 localizes in close proximity to vinculin-positive focal adhesions (arrows) as well as in a vesicular compartment (arrowheads) during focal adhesion assembly and at early time points following focal adhesion disassembly (Figure 7B). Although LC3-positive autophagosomes are present during focal adhesion reorganization, there is limited colocalization with either endogenous or GFP-tagged FIP200 (Figure 7C). Collectively, these data suggest that FIP200 may perform its distinct functions by directly targeting to focal adhesions to regulate downstream signaling events.

Phosphorylation of Src at tyrosine 416 is required for maximal kinase activity (Kmiecik and Shalloway, 1987) and is suggested to promote integrin-mediated adhesion associated with cell migration (Sanjay et al., 2001; Cary et al., 2002). Interestingly, both loss of FIP200 and Atg5 result in a dysregulation of Src phosphorylation at tyrosine 416 (pY416) in the context of Nocodazole induced FA reorganization (Figures 7D,E). The absence of both FIP200 and Atg5 result in elevated phospho-Src at basal levels and although there is a similar profile compared to wild-type during the Nocodazole washout assay, there remains elevated overall levels. Immunofluorescence microscopy revealed no colocalization of phospho-Src with LC3-positive autophagosomes during the time course of Nocodazole treatment and washout (Figures 7F,G). However, specific loss of Atg5 resulted in a retention of phospho-Src at focal adhesions, which was not apparent following FIP200 loss of function (Figures 7F,G). This phenotype was consistent with the phospho-FAK localization observed in Atg5 KO cells following fibronectin-induced cell spreading (Figure 3D). Overall, these data suggest a disruption in the activation status of Src following both FIP200 and Atg5 loss, but a specific retention of phospho-Src in focal adhesions following bulk autophagy disruption.

## Discussion

**FIGURE 8:**
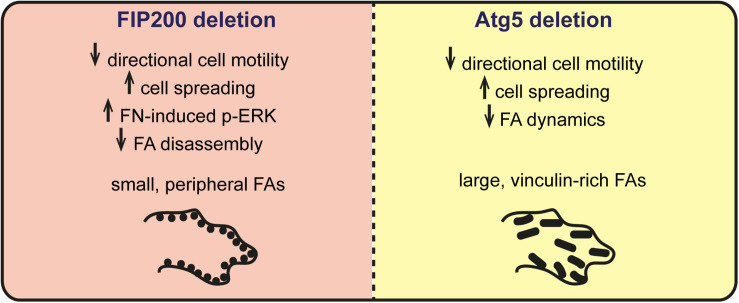
Summary of phenotypes associated with FIP200 or Atg5 loss of function. Loss of FIP200 in MCF10A cells results in small, peripheral focal adhesions (FAs), decreased directional cell motility, increased fibronectin-induced cell spreading, increased fibronectin-induced phospho-ERK (p-ERK) and a defect in FA disassembly. Loss of Atg5 in MCF10A cells results in decreased directional cell motility, increased fibronectin-induced cell spreading, and large, vinculin-rich FAs showing decreased dynamics.

Autophagy has been suggested to modulate focal adhesion organization and dynamics to regulate cell adhesion, migration, and survival (Sandilands et al., 2012a; Kenific et al., 2016a). It is currently thought that autophagy coordinates this by selectively degrading focal adhesion components, such as Src and paxillin, to trigger focal adhesion disassembly (Sandilands et al., 2012a; Sharifi et al., 2016). In this study, we aimed to determine whether the primary autophagy regulators Atg5 and FIP200 regulate cell motility and adhesion by a conserved mechanism. However, our results indicate that while loss of Atg5 and FIP200 both inhibit directional cell motility and enhance cell spreading on fibronectin in a similar manner, they have contrasting effects on focal adhesion composition, organization, and dynamics (Figure 8). Therefore, we suggest that while there may be an Atg5- and FIP200-dependent autophagy function to regulate focal adhesion turnover, additional autophagy-independent functions of each likely exist that differentially impact on focal adhesion organization.

Although both Atg5 and FIP200 loss of function affects directional cell motility, our results indicate that Atg5 KO cells are capable of eventual wound closure, while FIP200 are completely deficient. This disparity may be due to FIP200’s alternative mechanisms to regulate FAK activity (Abbi et al., 2002), but whether this is an autophagy-independent process is unknown. In relation to this, our results indicate that depletion of FIP200 leads to a reduction in FAK autophosphorylation, which negatively impacts on focal adhesion disassembly, which is not apparent in Atg5 null cells. This may indicate that FIP200’s role in FAK regulation is via an autophagy-independent function. However, the effect of FIP200 depletion on FAK phosphorylation conflicts with previous reports indicating that FIP200 acts as a negative regulator of FAK and the related protein Pyk2 (Ueda et al., 2000; Abbi et al., 2002). Therefore, further investigation will be required to determine whether these underlying differences are cell and context dependent.

Importantly, when we analyzed focal adhesion composition by quantifying the protein levels of FAK, vinculin, and paxillin in Atg5 KO and FIP200 KO cell lines, we detected contrasting effects on these individual focal adhesion components. These differences were supported by a distinct focal adhesion organization within the two paradigms, illustrated by large focal adhesions in Atg5 KO cells and small peripheral focal contacts in the FIP200 KO cells. In particular, the vinculin-positive focal adhesions in Atg5 KO cells were more robust and associated with actin stress fibers, which did not respond to microtubule destabilization. While in FIP200 KO cells, the focal adhesions were peripherally organized, which coincided with a cortical actin arrangement. In addition, these cells were able to stimulate significant focal adhesion assembly in response to microtubule destabilization, however, these adhesions were unable to disassemble back to steady state levels as observed for wild-type cells. We believe this defect in the disassembly process likely results from the inhibition in FAK autophosphorylation, since this is a prerequisite for focal adhesion breakdown (Hamadi et al., 2005) and is specific for FIP200 loss of function (Figures 5A–D). We therefore conclude that bulk autophagy inhibition alone has a discrete effect on focal adhesion organization and composition compared to FIP200 loss. These results are supported by findings in Atg5^–,–^ MEFs which have been shown to contain increased paxillin-positive adhesions and exhibit an enhancement in cell spreading (Kawano et al., 2017).

Interestingly, although there are noticeable differences in focal adhesion composition, organization and dynamics between Atg5 and FIP200 loss of function models, both exhibit a defect in directional cell motility and an enhancement in fibronectin-induced cell spreading. It is possible that in these cell lines there is enhanced membrane protrusion formation alongside an inability for polarization, which is required for directional cell motility. Importantly, we detect an activation of autophagy during fibronectin-induced cell spreading demonstrated by p62 accumulation and increased LC3 puncta, which suggests it plays an active role during this process. However, it should be noted that loss of FIP200 has a more pronounced effect on cell motility and spreading, suggesting there may be both autophagy-dependent and FIP200 specific roles during cell-ECM adhesion dynamics. In support of this, we demonstrate that FIP200 depletion results in the accumulation of phosphorylated FAK in intracellular vesicles, despite overall phospho-FAK being reduced in these cells. This contrasts with Atg5 KO cells, which retain phospho-FAK and phospho-Src at focal adhesions, suggesting a defect in their internalization and inactivation. In addition, FIP200 KO cells exhibit an enhancement in ERK phosphorylation in response to fibronectin adhesion, which is not apparent in Atg5 KO cells. Interestingly, it is known that active ERK is recruited to newly formed focal adhesions following integrin receptor engagement to facilitate focal adhesion assembly (Fincham et al., 2000). Thus, FIP200 loss may result in increased nascent focal adhesion assembly resulting in enhanced cell spreading due to a retention of active ERK at new adhesion sites. This is supported by our results indicating Nocodazole treatment in FIP200 KO cells causes a significant increase in focal adhesion assembly over wild-type cells.

FIP200 has been indicated to be a direct regulator of FAK activity, which our data supports in some respect. We therefore propose that FIP200 likely regulates FA maturation, whereby loss of its autophagy-dependent and -independent functions results in small focal contacts. This is indicated by an accumulation of nascent adhesions, supported by a distinct focal adhesion composition, resulting in a decreased ability to sustain directional motility due to a defect in maturation and stabilization of contact sites. Thus, our data suggests that FIP200 may play a role in nascent adhesion maturation or turnover, which is essential for the transfer to mature FAs required for traction force during cell migration (Schmidt et al., 1993). This contrasts with Atg5 KO cells which exhibit an accumulation of large ECM adhesions, rich in vinculin, indicative of mature FAs (Cox et al., 2003) and a retention of FAK-Src activation at focal adhesions (Figure 8). The impact of FIP200 loss of function on adhesion maturation and directional cell motility may be via its influence on the activity state and turnover of FAK, which is required for functions such as talin recruitment to enable adhesion maturation (Lawson et al., 2012). Future studies are required to determine the detailed mechanisms of FIP200 regulation of focal adhesion dynamics and whether there is cross-talk with the autophagy pathway during these processes. Importantly, our results suggest careful consideration needs to be taken when genetically targeting autophagy, as many essential regulators have autophagy-independent roles that may have wide-ranging cellular impacts.

## Materials and Methods

### Antibodies and Reagents

The V-ATPase inhibitor Bafilomycin A1 (ACROS Organics; A0360555) was used at a concentration of 100 nM. TGFβ1 (R&D Systems; 240B0020) was used at a concentration of 10 ng/ml. The antibodies used and their dilutions for western blot (WB) and immunofluorescence microscopy (IF) were as follows: FIP200/RB1CC1 rabbit polyclonal (Proteintech; 17250-1-Ab) WB 1:1000, IF 1:200; LC3B/MAP1LC3B rabbit polyclonal (Novus Biologicals; NB100-2220) WB 1:2000, IF 1:200; p62/SQSTM1 rabbit polyclonal (ProSci; 5449) WB 1:2000, IF 1:300; actin mouse monoclonal (612656) WB 1:3000, p-Akt ser473 rabbit monoclonal (560378) WB 1:800, fibronectin mouse monoclonal (610077) WB 1:2000, IF1:200; E-cadherin mouse monoclonal (610181) WB 1:4000, paxillin mouse monoclonal (610619) WB 1:2000, and FAK pY397 (61172) WB 1:1000, IF 1:200 were purchased from BD Transduction Laboratories; phospho-p44/42 MAPK (Erk1/2) (137F5) rabbit monoclonal (Cell Signalling Technology) WB 1:1000; FAK mouse polyclonal (BioLegend; 603801) WB 1:1000; vinculin mouse monoclonal (EMD Millipore; MAB3574) WB 1:2000, IF 1:200; Atg5 rabbit polyclonal (Cell Signalling; 2630S) WB 1:3000; GAPDH rabbit polyclonal (ProSci; 3781) WB 1:1000.

### Plasmids

The pME18s-HA-hFIP200 was a gift from Noboru Mizushima (Addgene plasmid #24303^1^; RRID:Addgene_24303). The FIP200 cDNA was PCR cloned into the pEGFPc2 vector using *Eco*RI and *Kpn*I restriction sites. We used the bicistronic Cas9/sgRNA mammalian expression vector, pX459 [pspCas9(BB)-2A-Puro] (Addgene; 48139), to create knockout cell lines. The vector was digested with Bbsl ligated to the annealed 20 base pair long oligos encoding the FIP200 and ATG5 gRNAs. The FIP200 gRNA 5′-TATGTATTTCTGGTTAACAC-3′ targeted exon 3 and the Atg5 gRNA 5′-AAGATGTGCTTCGAGATGTG-3′ targeted exon 2. Positive clones were screened by sequencing with a U6 promoter forward primer to confirm correct insertion of each gRNA.

### Cell Culture

MCF10A cells were cultured in DMEM/F-12 + Glutamax supplemented with 5% Horse serum (Thermo Fisher), 20 ng/ml EGF (Sigma-Aldrich; E9644), 0.5 mg/ml Hydrocortisone (Sigma-Aldrich; H0888), 100 ng/ml Cholera Toxin (Sigma-Aldrich; C8052), 10 μg/ml Insulin (Sigma-Aldrich; I9278) and 1% Penicillin/Streptomycin. Cell transfections with cDNA were performed using FuGene^®^ 6 transfection reagent (Promega) according to the manufacturers protocol. Cells were harvested for analysis 24 h post-transfection.

For generation of knockouts, cells within a 6-well plate were transfected with 2 μg of each pX459 construct and 6 μL FuGene^®^ 6 transfection reagent diluted in Opti-MEM^®^ media. 24 h post transfection, cells were washed and replenished with growth media containing 1.5 μg/mL puromycin. Cells were selected for 48 h, prior to replenishment in growth media and single cell cloning. Clonal cell lines were isolated and screened by western blot for the presence of knockout.

### Scratch Wound Assay

MCF10A cells were plated onto a 6-well plate in growth medium at 80–90% confluency. The following day media was replaced with EGF free medium and cells were starved of EGF over-night. The next day, using a p100 pipette tip, a wound was made by scratching a vertical line in the center of the well. Cells were then washed thoroughly with PBS to ensure the removal of all un-adhered cells. Cells were then stimulated with EGF via incubating cells with MCF10A growth medium containing EGF. 30 min post-scratch, images were taken (EVOS XL Core Cell Imaging System, Thermo Fisher Scientific) at multiple regions of the wound and this was recorded as the 0 h time point. Subsequent images were then taken at the same spot at the appropriate time points (0, 6, 12, and 24 h). Wound area was measured using ImageJ software and wound closure was based on difference in area of the wound region from 0 h (0% wound closure). Four wound regions were analyzed per each individual experiment and wound closure values are mean ± SEM from 3 individual experiments. Two-way ANOVA with Bonferroni correction was carried out for statistical analysis using Graphpad Prism 6.07.

### Fibronectin Spreading Assay

Twelve well plates or glass coverslips were coated with 10 μg/mL fibronectin (EMD Millipore; FC010) diluted in PBS and left to incubate overnight at 37°C. The following day fibronectin was removed, and the plates were washed with PBS and blocked for 3 h with DPBS containing 3% BSA. The BSA block was removed and wells were washed with PBS 3 times prior to cell plating. MCF10A cells were trypsinised and pelleted via centrifugation and resuspended in EGF free media. Cells were left in suspension for 1 h at 37°C in a humidified atmosphere of 5% CO_2_ to allow recovery of cell surface receptors. Following this, cells were plated at 70% confluency onto fibronectin coated dishes or coverslips. For the 0 h time point for western blot analysis, the remainder of the cell suspension was pelleted, washed with PBS, and lysed. Cells plated on fibronectin were left to spread for 30, 60, 120, 180, and 240 min at 37°C prior to cell lysis or fixation. Several bright-field images were also taken of the cells to observe phenotypic changes during cell spreading at these time points immediately prior to lysis or fixation (EVOS XL Core Cell Imaging System, Thermo Fisher Scientific). 4 images per time point were taken and for each image 25 cells were measured for area size using ImageJ software, to get a pixel area value for each cell from 100 cells per time point. Two-way ANOVA with Bonferroni correction was applied to cell area data for statistical analysis using Graphpad Prism 6.7.

### Western Blot

Cells were washed with ice cold PBS and lysed with ice cold Triton-SDS lysis buffer (1% Triton X-100, 0.1% SDS, 150 mM NaCl, 10 mM Tris HCl pH 7.4, 5 mM EDTA, 10 mM NaF, and cOmplete mini EDTA protease inhibitor cocktail, Roche). Cell lysates were collected and clarified by centrifugation at maximum speed in a microfuge for 10 min at 4°C. Soluble cell extracts underwent a protein assay using Pierce BCA protein assay kit (Thermo Fisher Scientific) to enable optimized protein loading of lysates. These were then separated by SDS-polyacrylamide gel electrophoresis (SDS-PAGE), alongside a molecular weight protein marker (EZ-Run prestained Rec Protein Ladder (Thermo Fisher). After separation, proteins were transferred to Immobilon-FL PDVF membrane (Millipore) using a wet protein transfer method (350 mA, 1 h). Membranes were then incubated in blocking buffer (5% Milk powder or 3% BSA in TBS) for 1 h at room temperature. Membranes were incubated with primary antibody in block at 4°C, overnight. Prior to incubation with secondary antibody, membranes were washed 4 min × 5 min in TBS, 0.1% Tween-20. Blots were incubated with secondary antibody at room temperature for 2 h in TBS, 0.1% Tween-20. Secondary antibodies used were IRDye^®^ 800 anti-rabbit and IRDye^®^ 680 anti-mouse, both at 1:5000 (Li-Cor^®^). Subsequently, blots were washed 4 min × 5 min with TBS, 0.1% Tween-20, and then a final wash with TBS. Blots were scanned using the Li-COR Odyssey Infrared Imaging system. Band densitometric values were attained using Li-COR Image studio Lite Ver 5.2 and were normalized against a loading control.

### Immunofluorescence Microscopy

Cells were seeded onto glass coverslips placed inside 6-well plates. Cells were fixed with 4% formaldehyde in PBS for 20 min, and permeabilized with 0.02% Triton X-100 in PBS for 2 min. Cells were blocked with 1% BSA in PBS for 20 min, and subsequently incubated with primary antibody diluted in 1% BSA for 1.5 h at room temperature. Cells underwent a PBS wash prior to incubation with secondary antibody for 45 min. Secondary antibodies used for visualization of immunofluorescence staining were either Alexa Fluor 488 goat anti-mouse (A-11001), 488 goat anti-rabbit (A-11008), 568 goat anti-mouse (A-11004), or 568 goat anti-rabbit (A-11011) (Life Technologies, 1:200). Images were obtained using a Zeiss Axioplan epifluorescence microscope using either a 63× or 100× oil immersion objective. Images were imported into Adobe Photoshop CS6 and adjusted for brightness and contrast.

For Nocodazole washout assay, cells were serum starved for 18 h prior to treatment with 10 μM Nocodazole (Sigma, M1404) for 4 h. Following this, cells were washed 2× to washout Nocodazole and incubated in growth media allowing recovery of the microtubule cytoskeleton. Cells were harvested for immunofluorescence microscopy at indicated time points and images acquired using a 63× objective. Images were imported into ImageJ, thresholded, a rolling ball background subtraction was performed, and converted to binary. A region of interest was placed around each cell and the integrated density of focal adhesion pixel area was calculated. Quantitation of vinculin-positive focal adhesion pixel area was performed on 5 random fields of view, comprised of 10–30 cells per experiment, from 2 to 3 independent experiments. A two-way ANOVA was performed followed by Bonferroni multiple comparison test.

Quantitation of p62, LC3, and FAK pY397 puncta was performed on images acquired using a 63× objective on a Zeiss Axioplan upright microscope. Images were imported into ImageJ, thresholded, and a rolling ball background subtraction was performed to ensure quantitation of only those spots exhibiting a signal above the cytosolic background. For p62, puncta from 100 individual cells across each group were quantified and for LC3, 13–15 individual cells across each group were quantified from 3 independent experiments. Data are represented as the average number of p62 or LC3 puncta per cell. For FAK pY397 quantitation, between 30 and 34 cells per group were quantified from 2 independent experiments. Data are represented as the number of FAK pY397 puncta per cell, where each data point indicates a cell.

## Data Availability Statement

The datasets generated for this study are available upon reasonable request to the corresponding author.

## Author Contributions

EA designed and performed the experiments and analyzed the data. DT conceived the study, designed the experiments, analyzed the data, and wrote the manuscript. Both authors contributed to the article and approved the submitted version.

## Conflict of Interest

The authors declare that the research was conducted in the absence of any commercial or financial relationships that could be construed as a potential conflict of interest.

## Footnotes

1 http://n2t.net/addgene:24303

## Supplementary Material

Ensure that any supplementary material is correctly published at this link: https://www.frontiersin.org/articles/10.3389/fcell.2020.00733/full#supplementary-material
