# Supplementary figures and images for: Loss of the Essential Autophagy Regulators FIP200 or Atg5 Leads to Distinct Effects on Focal Adhesion Composition and Organization
Source: Front Cell Dev Biol. 2020 Aug 4;8:733. doi: 10.3389/fcell.2020.00733 (PMC7417463; doi:10.3389/fcell.2020.00733)

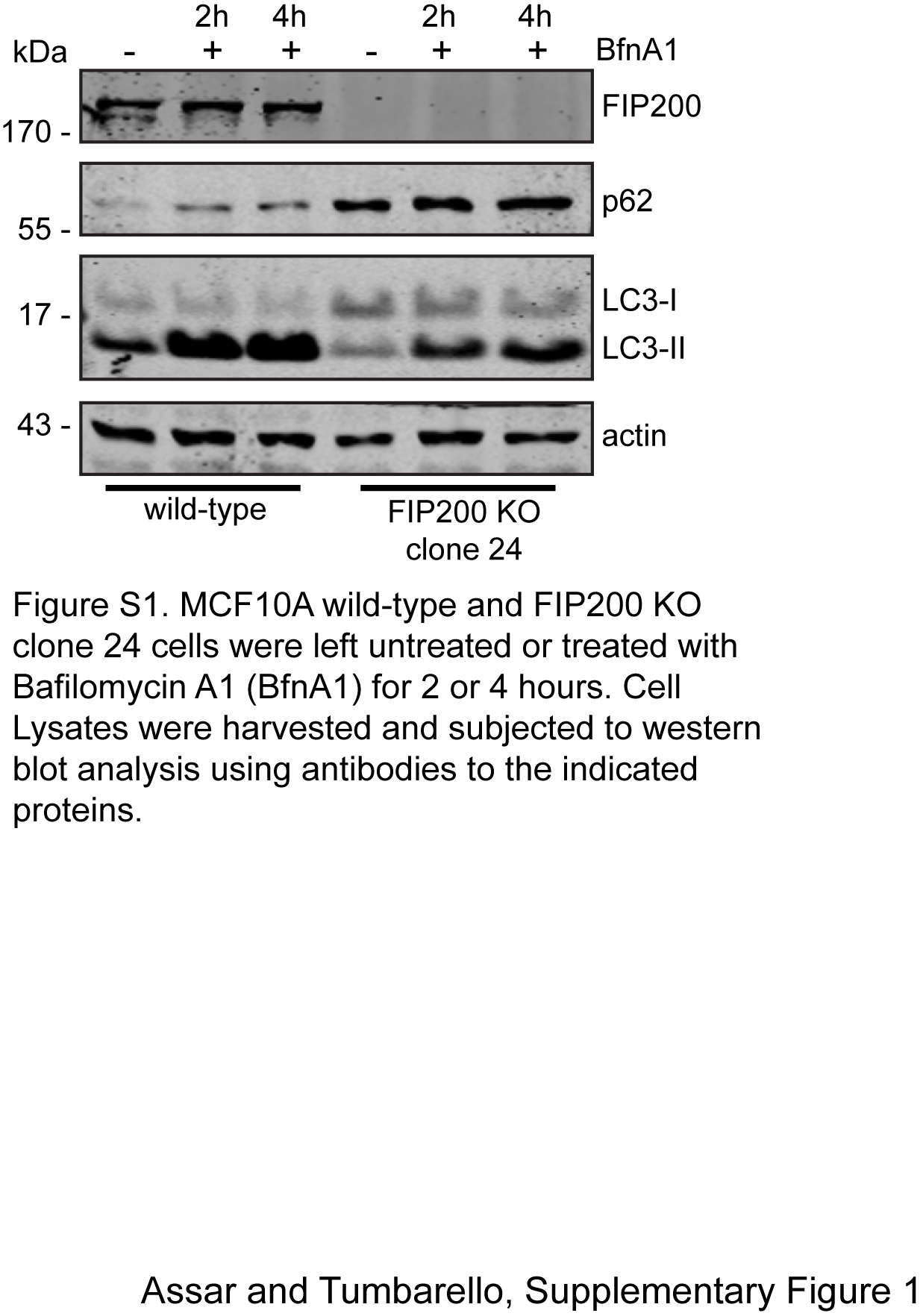

Supplement: Supplementary file 1 [file Image_1.TIF]

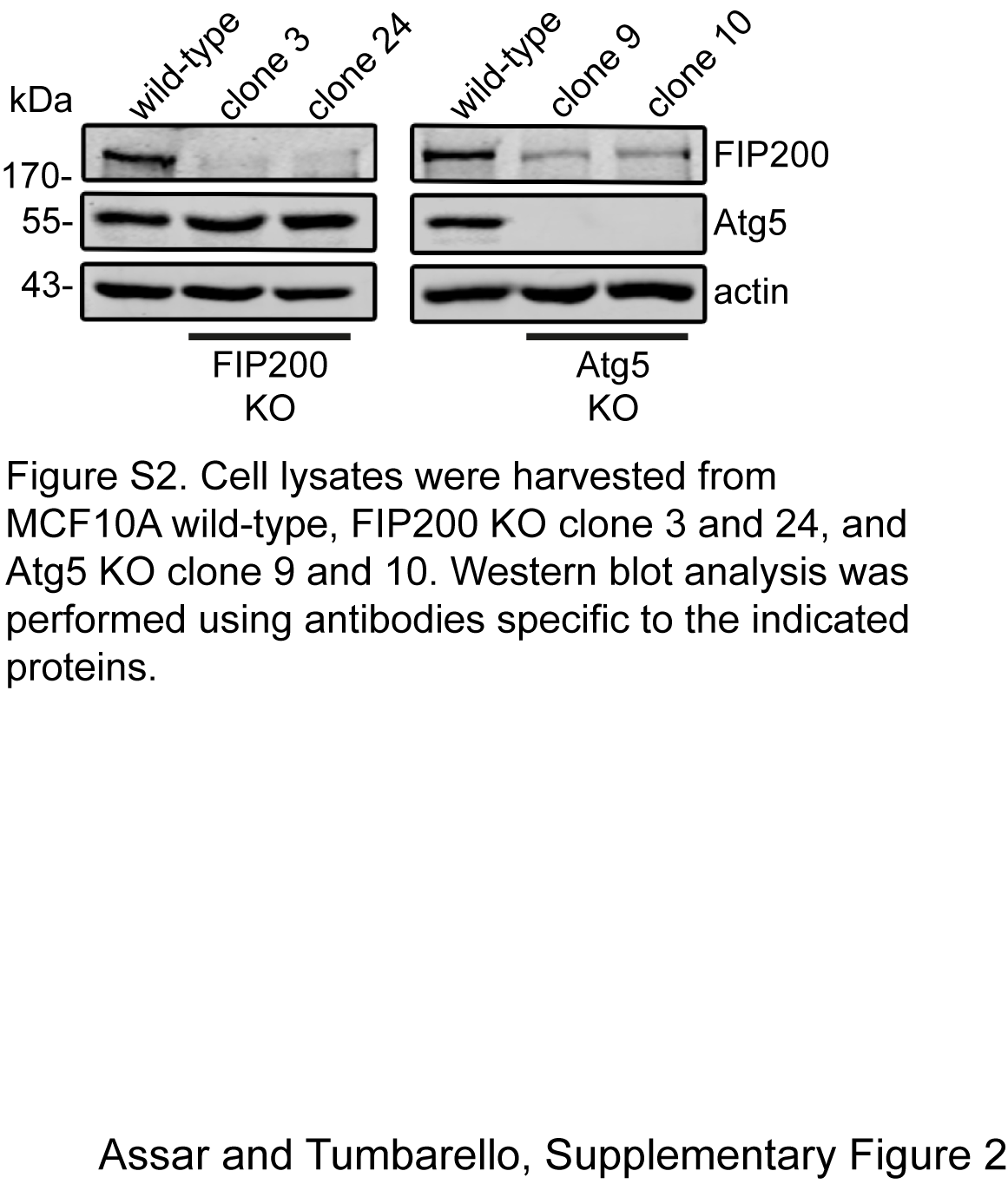

Supplement: Supplementary file 2 [file Image_2.TIF]
